# Drug cue reactivity involves hierarchical instrumental learning: evidence from a biconditional Pavlovian to instrumental transfer task

**DOI:** 10.1007/s00213-017-4605-x

**Published:** 2017-04-15

**Authors:** Lorna Hardy, Chris Mitchell, Tina Seabrooke, Lee Hogarth

**Affiliations:** 1 School of Psychology, University of Exeter, Washington Singer Building, Perry Road, Exeter, EX4 4QG UK; 2 School of Psychology, Plymouth University, Portland Square, Drake Circus, Plymouth, PL4 8AA UK

**Keywords:** Cue reactivity, Binary associations, Hierarchical learning, Alcohol problems

## Abstract

**Rationale:**

Drug cue reactivity plays a crucial role in addiction, yet the underlying mechanisms are poorly understood. According to the binary associative account, drug stimuli retrieve an expectation of the drug outcome, which, in turn, elicits the associated drug-seeking response (S-O-R). By contrast, according to the hierarchical account, drug stimuli retrieve an expectation that the contingency between the drug-seeking response and the drug outcome is currently more effective, promoting performance of the drug-seeking response (S:R-O).

**Methods:**

The current study discriminated between these two accounts using a biconditional Pavlovian-to-instrumental transfer (PIT) task with 128 alcohol drinkers. A biconditional discrimination was first trained in which two responses produced alcohol and food outcomes, respectively, and these response-outcome contingencies were reversed across two discriminative stimuli (SDs). In the PIT test, alcohol and food cues were compounded with the two SDs to examine their impact on percent alcohol choice in extinction.

**Results:**

It was found that alcohol and food cues selectively primed choice of the response that earned that outcome in each SD (*p* < .001), and this effect was associated with participants’ belief that cues signalled greater effectiveness of that response (*p* < .0001).

**Conclusions:**

The alcohol stimulus could not have selectively primed the alcohol-seeking response through binary S-O-R associations because the drug outcome was equally associated with both responses. Rather, the alcohol stimulus must have retrieved an expectation that the response-alcohol contingency available in the current context was more likely to be effective (S:R-O), which primed performance of the alcohol-seeking response.

## Introduction

Drug cue reactivity is a central construct in addiction research, and there have been numerous attempts to elucidate the underlying learning mechanisms (e.g. Carter and Tiffany 1999). Drug cue reactivity was originally attributed to the formation of a direct association between the stimulus and the response (Wikler 1984), but later theories accepted that drug cues might elicit expectations of the drug, which drive drug-seeking behaviour (Stewart et al. 1984). Several sources of evidence are consistent with this latter view. First, drug conditioning studies have found that drug-paired conditioned stimuli (CSs) only elicit craving and drug consumption if participants possess knowledge of the predictive relationship between the CSs and the drug (Hogarth and Duka 2006). More decisively, conditioned craving to CSs can be immediately established by instructions stating that the CS predicts drug availability, and abolished by instructions stating that the CS no longer predicts drug availability (Dols et al. 2000; Field and Duka 2001). Such instruction effects on human non-drug conditioning have been extensively reported (Mitchell et al. 2009). Thus, drug expectancies appear to contribute causally to drug cue reactivity.

The Pavlovian-to-instrumental transfer (PIT) procedure provides a key method for studying the role of drug expectancies in drug-seeking behaviour. In a typical human drug PIT design, participants undergo instrumental training in which one response (R1) earns a drug reward outcome (O1), and another response (R2) earns a food outcome (O2) (R1-O1, R2-O2) (Hogarth et al. 2007). In a separate phase, participants learn that two Pavlovian stimuli differentially predict those same outcomes (S1-O1, S2-O2). In the transfer test, the Pavlovian stimuli are presented while participants freely choose between the two responses in extinction (S1:R1/R2, S2:R1/R2). It has been found that each cue selectively augments choice of the response that earns the same (congruous) outcome (S1:R1 > R2, S2:R1 < R2) (Hogarth et al. 2007). The capacity of the drug stimulus to selectively prime the drug-seeking response cannot be attributed to the formation of an S-R association (habit learning) because the Pavlovian stimulus and the instrumental response are trained in separate stages and so are never paired prior to testing. Rather, to explain this effect, the drug stimulus must retrieve an expectation (or representation) of the drug outcome with which it was paired, to specifically prime the response that was paired with the same outcome.

There are two variants of this expectancy-based account of PIT. The S-O-R account argues that the PIT effect is driven by a chain of binary associations between stimuli, outcomes and responses (de Wit and Dickinson 2009). Specifically, in the Pavlovian phase, each stimulus forms a binary association with (and can elicit an expectation of) its associated outcome (S1-O1, S2-O2). Similarly, in the instrumental training phase, each response forms a binary association with its associated outcome (R1-O1, R2-O2). Crucially, these R-O links are bidirectional such that an S-elicited expectation of a particular O can elicit the associated R through the chain of S-O-R links. Thus, each S selectively primes one R through an expectation of the outcome, shared by both the S and R.

The hierarchical account, by contrast, argues that the PIT effect is driven by stimuli retrieving an expectation (or representation) of which R-O relationship is currently in force (S:R-O) (Dickinson 1997; Rescorla 1991). In the context of cue reactivity, the presence of particular drug stimuli (e.g. a bar or pub open sign) retrieves an expectation that a particular drug-seeking response (walk in and buy a drink) is likely to be effective in producing the drug (a drink), raising the propensity to perform this response. To explain the PIT effect, the hierarchical account argues that S:R-O relations are learned in both the Pavlovian and instrumental phases. In the Pavlovian phase, S1 and S2 signal that a common tacit response (e.g. hopper entry, saccade, approach) produces access to O1 and O2, respectively. By contrast, in the instrumental phase, a common contextual stimulus signals that R1 and R2 produce access to O1 and O2, respectively. The PIT effect in the transfer test is produced by a combination of (inference between) the S:R-O relations acquired in these two stages. That is, S1 is inferred to signal that the R1-O1 contingency is in force, whereas S2 is inferred to signal that the R2-O2 contingency is in force. These expectancies drive performance of the viable response. In other words, each stimulus elicits a goal-directed expectation that the R-O contingency for the shared O is more likely to be effective, which primes performance of that R (Hogarth et al. 2014; Seabrooke et al. 2015).

The binary versus hierarchical explanations of PIT can be distinguished using a biconditional discrimination task. This task has demonstrated that animals are capable of hierarchical learning (e.g. Bradfield and Balleine 2013; Colwill and Rescorla 1990; Trask and Bouton 2014), but has rarely been used in humans (Declercq and De Houwer 2009). The current study employed a novel human biconditional PIT task with alcohol and food outcomes to test whether drug stimulus control of drug-seeking is underpinned by binary or hierarchical learning. In the biconditional training phase, participants learned that in one discriminative stimulus (SD1), R1 earned alcohol O1, and R2 earned food O2 (SD1: R1-O1, R2-O2). These response-outcome contingencies were reversed in the second SD (SD2: R1-O2, R2-O1). In the transfer test, an alcohol or food image was presented together with each SD. The purpose of this phase was to test whether the alcohol and food stimuli could selectively prime the response which earned the congruous outcome in the current SD (a biconditional PIT effect).

This biconditional PIT effect could not be explained by binary S-O-R associations because all binary associations between SDs, outcomes and responses are equated by the biconditional schedule (the original purpose of this procedure; Rescorla 1991). That is, the S-O-R account predicts that when the alcohol stimulus is presented at test it will elicit an expectation of the alcohol outcome (S-O). However, because this outcome has been equally associated with both responses, it should prime both responses equally through the O-R link, creating no selective choice of the response which earns the alcohol outcome in the current SD (no biconditional PIT effect). The same is true for the food stimulus. By contrast, the hierarchical account anticipates that alcohol and food stimuli will produce a biconditional PIT effect on the grounds that these stimuli retrieve knowledge of hierarchical S:R-O contingencies, i.e. knowledge of which response produces the congruous outcome in the current SD, because they are functionally similar to (have acquired equivalence with) the SD used in the training stage (Hall et al. 2003). Arguably, the alcohol and food stimuli elicit an expectation that the response which earns the congruous outcome in the current SD is more likely to be reinforced, which selectively primes that response. This claim was further tested by asking participants after the PIT test to rate the extent to which they thought that the alcohol and food stimuli signalled that the congruous response was more likely to be reinforced. A correlation between these expectations and the biconditional PIT effect would support the claim that the biconditional PIT effect is underpinned by hierarchical knowledge of S:R-O relations. Evidence for a hierarchical account of drug cue reactivity would have implications for treatment strategy.

## Method

### Participants

One hundred and twenty-eight students who reported drinking at least occasionally (50% male) were recruited at the University of Exeter. There were no other inclusion criteria. Ethical approval was obtained from the University of Exeter Research Ethics Committee.

### Questionnaires

Participants reported age, gender and alcohol use/alcohol-related problems in the Alcohol Use Disorders Identification Test (AUDIT) (Babor et al. 2001).

### Biconditional training

Participants were instructed that “In this task, you can earn beer and chocolate to take away at the end. In each trial, press the left or right key to win a point for these rewards. Different arrow shapes indicate which key earns which reward. It is your task to learn this. Press any key to begin”. Participants were shown the alcohol reward (a 275-ml bottle of Becks) and the food reward (a 45-g bar of Dairy Milk), and these remained in sight. This was a deception. All participants were given a small chocolate bar at the end of testing.

Sequential training established the biconditional contingencies (Table 1). The first block of eight trials began with SD1, a particular arrow symbol (black or blue) pointing in both directions signalling that either a left or right key press response could be made. Participants were free to press either the left or right arrow keyboard key. Pressing a key presented the outcome text “You earn beer” (O1) or “You earn chocolate” (O2) below the arrow symbol for 1 s prior to a random intertrial interval (ITI) of 350–750 ms. SD1 signalled that response 1 (R1) earned alcohol and response 2 (R2) earned food (SD1: R1-O1, R2-O2). These response-outcome contingencies were deterministic; that is, they produced their relevant outcome with 100% probability on a fixed ratio 1 schedule. In the next block of eight trials, the arrow symbol SD2 was presented, which signalled that the reverse R-O mappings were in effect, i.e. SD2: R1-O2, R2-O1. Whether black or blue arrow symbols functioned as SD1 or SD2 in the two blocks was counterbalanced between subjects, as well as the left/right responses that functioned as R1 and R2. Following these 16 trials, participants reported their knowledge of the biconditional contingencies in four questions in which SD1 and SD2 were presented twice, along with the questions (in random order): “When this arrow was present, which key earned [beer/chocolate] the LEFT or RIGHT key?” Participants were deemed to have acquired knowledge of the biconditional contingencies when they got all four questions correct, and sequential training blocks continued until this criterion was met. Participants then experienced intermixed training, in which SD1 and SD2 trials were randomly intermixed across each set of 16 training trials. Training continued until all four contingency questions were correctly answered.

**Table 1:** The arrangement of the training, test and expectancy phases

Biconditional training	Transfer test	Expectancy test
SD1: R1-O1, R2-O2 SD2: R1-O2, R2-O1	AlcoholS + SD1: R1/R2 AlcoholS + SD2: R1/R2 FoodS + SD1: R1/R2 FoodS + SD2: R1/R2 BlankS + SD1: R1/R2 BlankS + SD2: R1/R2	AlcoholS/FoodS: “When this picture was presented, to what extent did you think that the [beer/chocolate] key was more likely to be rewarded?”

SD1 and SD2 were blue and black arrow keys which signalled the reversal of two response-outcome (R-O) contingencies. R1 and R2 were left or right keyboard arrow presses. O1 was beer points, and O2 was chocolate points. AlcoholS was a picture of beer, FoodS was a picture of chocolate, and BlankS was a grey square

### Transfer test phase

Participants were instructed: “In this part of the task, you can earn beer and chocolate in the same way as before. However, you will only be told how much you have earned at the end of the experiment. Press any key to begin”. This phase was conducted in nominal extinction to test the effect of cues in the absence of feedback from outcomes (Table 1). In each trial, the arrow symbol SD1 or SD2 was displayed with a picture of either alcohol (two beer glasses being tapped together), food (close-up of chocolate chunks) or a blank grey image, located above the arrow symbol. Participants then made a left or right key press but received no feedback about the outcome earned and, instead, the ITI of 350–750 ms launched before the next trial. There were 48 transfer trials, comprising 4 cycles of 12 trials, in which the two arrow symbols (SD1, SD2) were presented with each of the three stimuli (alcohol, food and blank) twice for each combination. Alcohol and food images were expected to augment choice of the arrow key which produced the congruous outcome in that context.

### Expectancy scores

Participants’ expectations that stimuli signalled effective R-O relations were then measured in two questions. Participants were told “We would now like to examine your thoughts about the beer and chocolate pictures. Please think carefully about your answers. Press any key to begin”. Participants were presented with the alcohol and food stimuli individually in separate trials in random order. Upon presentation of the alcohol stimulus, they were asked: “When this picture was presented, to what extent did you think that the beer key was more likely to be rewarded? Press a key from 1 to 7”, with a Likert scale from 1 (not at all) to 7 (very much). Upon presentation of the food stimulus, they were asked: “When this picture was presented, to what extent did you think that the chocolate key was more likely to be rewarded? Press a key from 1 to 7”. Finally, participants’ knowledge of the biconditional contingencies was tested as before.

### Analysis

Analysis of variance (ANOVA) first tested whether the alcohol and food stimuli increased choice of the response for the congruous outcome, collapsed across the two SDs. An analysis of covariance (ANCOVA) then tested whether the biconditional PIT effect increased with mean expectancies that stimuli signalled greater efficacy of the corresponding response (mean expectancy scores in the beer and chocolate stimulus were collapsed because they were so highly correlated, *r* = .74, *p* < .001). This effect would suggest that cue reactivity is driven by knowledge of hierarchical relations. A similar ANCOVA was run to determine if the biconditional PIT effect varied with alcohol use/problems, indexed by the AUDIT.

## Results

### Participants

Of 128 participants, eight participants reported inaccurate knowledge of the biconditional contingencies following the transfer test and were excluded (Hogarth et al. 2007; Trick et al. 2011). One participant was excluded for requiring an outlying number of sequential training blocks to acquire contingency knowledge (ten 16-trial blocks). The mean for the remaining 119 participants (54% male) was 1.3 blocks (range = 1–4). The mean number of intermixed blocks required to report accurate knowledge was 1.2 (range 1–5). The remaining sample had a mean age of 20.7 (range = 19–38) and a mean AUDIT score of 13.4 (range = 1–30).

### Transfer test

**Fig. 1:**
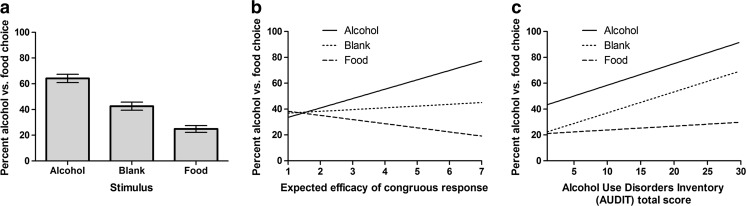
**a** Bar chart showing the mean percent choice of alcohol in alcohol, blank, and food stimulus conditions of the transfer test. **b** Regression slopes plotting the percent choice of alcohol in the alcohol, food, and blank stimuli of the transfer test against the mean expectancy score (1–7) that stimuli signalled greater efficacy of the congruous response-outcome relation. **c** Percent choice of alcohol in the alcohol, food, and blank stimuli of the transfer test plotted against the alcohol use/alcohol-related problems (AUDIT) scores

Figure 1a shows the percent choice of alcohol over food in alcohol, food and blank stimulus trials, collapsed across SD1 and SD2. ANOVAs on these data yielded a significant main effect of stimulus (*F*(2,236) = 70.71, *p* < .001, eta^2^ = .37), where alcohol differed from food (*F*(1,118) = 99.15, *p* < .001, eta^2^ = .46) and blank (*F*(1,118) = 44.55, *p* < .001, eta^2^ = .27) and food differed from blank (*F*(1,118) = 45.90, *p* < .001, eta^2^ = .28). The extent to which alcohol and food stimuli primed their corresponding responses relative to blank trials was comparable (*F*(1,118) = .77, *p* < .38, eta^2^ = .01). Thus, cues were highly effective in promoting the response which produced the congruous outcome in the discriminative context (SD1 and SD2), supporting a hierarchical account of cue reactivity.

Figure 1b shows that the biconditional PIT effect varied with expectations that cues signalled greater efficacy of the corresponding response. ANCOVA on these data revealed a significant interaction between stimulus and expectancy (*F*(2,234) = 16.79, *p* < .0001, eta^2^ = .13) and no main effect of expectancy (*F*(1,117) = 1.84, *p* = .17, eta^2^ = .02), indicating that overall alcohol choice did not increase with expectancy. The interaction between stimulus and expectancy was reliable when the model was restricted to alcohol and food trials (*F*(1,117) = 24.92, *p* < .0001, eta^2^ = .18), alcohol and blank trials (*F*(1,117) = 10.65, *p* = .001, eta^2^ = .08) and food and blank trials (*F*(1,117) = 9.60, *p* = .002, eta^2^ = .08). These findings suggest that cue reactivity is associated with knowledge of hierarchical relations.

Figure 1c shows that the biconditional PIT effect varied with alcohol use/problem (AUDIT) scores. There was a main effect of AUDIT (*F*(1,117) = 13.23, *p* < .001, eta^2^ = .10), indicating that alcohol use/problems were associated with greater alcohol choice overall. There was also a significant interaction between stimulus and AUDIT (*F*(2,234) = 5.04, *p* = .007, eta^2^ = .04), suggesting that the PIT effect varied with alcohol use/problems. However, the interaction between stimulus and AUDIT was not reliable when the model was restricted to alcohol and blank trials (*F*(1,117) = 0.01, *p* = .93, eta^2^ < .01), suggesting the alcohol PIT effect is constant across alcohol use/problems. By contrast, the interaction between stimulus and AUDIT was reliable when the model was restricted to food and blank trials (*F*(1,117) = 12.37, *p* = .001, eta^2^ = .10) and alcohol and food trials (*F*(1,117) = 5.51, *p* = .02, eta^2^ = .05), suggesting that the food PIT effect was compressed in low-dependent individuals because baseline food responding in blank trials was near maximal. Finally, AUDIT and expectancy scores were not significantly correlated (*r* = .09, *p* = .33).

## Discussion

The current study tested whether the capacity of alcohol cues to specifically promote alcohol-seeking behaviour is driven by binary S-O-R links or hierarchical S:R-O knowledge, using a biconditional PIT task. A biconditional discrimination was trained in which two SDs signalled the reversal of two R-O contingencies for alcohol and food outcomes, respectively (SD1: R1-O1, R2-O2. SD2: R1-O2, R2-O1). The transfer test found that alcohol and food stimuli presented with these SDs selectively primed performance of the response which earned the congruous outcome in each SD. This biconditional PIT effect cannot be explained by the S-O-R account because the binary associations between SDs, Os and Rs were all equivalent in the biconditional schedule. Specifically, because the alcohol and food outcomes have equal binary associations with both responses, the S-O-R account anticipates that the retrieval of an alcohol outcome expectancy by the alcohol stimulus would activate both Rs equally, producing no preferential selection between the two responses (the same is true for the food stimulus). Rather, for the alcohol and food stimuli to have selectively primed the congruous response, they must have retrieved hierarchical knowledge of which response produced that outcome in each SD (S:R-O). The finding that the magnitude of the PIT effect increased with participants’ expectations that alcohol and food stimuli signalled greater effectiveness of the congruous response supports the view that this effect is underpinned by hierarchical knowledge of S:R-O relations.

Several other findings support the hierarchical account of PIT. First, PIT effects are larger when R-O contingencies are partially reliable (33%) compared to fully reliable (100%) (Cartoni et al. 2015). The S-O-R theory anticipates the opposite finding because the O-R link is weaker in the unreliable condition and so should produce a smaller PIT effect. By contrast, the hierarchical account anticipates this finding because PIT effects should be greater when cues resolve uncertainty about the effectiveness of R-O contingencies. Second, PIT effects are generally larger with cues that have been trained as SDs compared to Pavlovian stimuli (Rescorla 1994; Troisi 2006). The S-O-R account predicts the opposite finding because discriminative training (S:R-O) should lead to overshadowing by the R, producing a weaker S-O link compared to Pavlovian training. By contrast, the hierarchical account anticipates this finding because stimuli that have been trained as SDs initially should be more readily treated as SDs in the PIT test (Hall et al. 2003). Finally, PIT effects are extinguished more rapidly if stimuli undergo discriminative extinction where the S signals that the R-O relation is not in force, compared to Pavlovian extinction where the S signals that the O will not occur (Delamater 1996; Gámez and Rosas 2005; Hogarth et al. 2014; Rescorla 1992; Rosas et al. 2010). Again, the S-O-R account predicts the opposite finding because Pavlovian extinction should more readily degrade the S-O link. In contrast, the hierarchical account anticipates this finding because discriminative extinction degrades the hierarchical S:R-O relations which underpin the PIT effect. Finally, the PIT effect can be abolished by verbal instructions that stimuli do not signal which response is more effective, or created by instructions stating that stimuli signal which response is more likely to be effective, suggesting that hierarchical knowledge of S:R-O relations is sufficient to drive the PIT effect (Hogarth et al. 2014; Seabrooke et al. 2015). However, it should be noted that although hierarchical knowledge underpinned the current biconditional PIT effect, it remains possible that simpler associative structures, such as S-R habit learning or binary S-O-R learning, could play a role in cue reactivity when biconditional contingencies are not in effect, and the current study cannot rule out this possibility.

The hierarchical account has implications for the treatment of cue reactivity. Studies have attempted to extinguish drug-seeking by means of Pavlovian extinction, where drug cues are presented without drug consumption, or instrumental extinction, where mock drug-taking does not produce drug reinforcement. Although these procedures reduce cue-elicited craving in the laboratory (Conklin and Tiffany 2002; Price et al. 2010; Xue et al. 2012), they do not abolish PIT effects (Delamater 1996; Hogarth et al. 2014; Rosas et al. 2010) or produce long-term improvements in abstinence (Conklin and Tiffany 2002). The hierarchical account anticipates these clinical failures because extinguishing binary S-O and R-O relations leaves hierarchical S:R-O relations intact. One might argue, therefore, that interventions should seek to degrade hierarchical knowledge using discriminative extinction training procedures (S:R-no O). These procedures are more effective at abolishing PIT in the laboratory (Delamater 1996; Gámez and Rosas 2005; Hogarth et al. 2014; Rescorla 1992; Rosas et al. 2010). However, the more intractable problem is that extinction learning generalises poorly between contexts (Collins and Brandon 2002; Thewissen et al. 2006), and there is no evidence that discriminative extinction would be any less susceptible to this problem. A possible solution could be the implementation of discriminative extinction training in the user’s natural environment with ecologically valid stimuli and responses. However, clients’ knowledge that bars and pubs signal the viability of alcohol-seeking behaviour is veridical with environmental contingencies and may not be susceptible to modification by cognitive behaviour therapy or gamified tasks. Psychologists might therefore be tempted to abandon retraining of cue reactivity in the natural environment and instead focus on minimising the pervasiveness of environmental drug cues by evaluating plain packaging policy (Hogarth et al. 2015) or the regulation of advertising (Jernigan et al. 2017), for example.

AUDIT scores were not associated with the alcohol PIT effect: the extent to which the alcohol stimulus primed alcohol-seeking above the blank condition. Such null associations between drug PIT and severity of drug use/problems have been found previously for alcohol (Garbusow et al. 2014; Martinovic et al. 2014) and tobacco (Hogarth 2012; Hogarth and Chase 2011, 2012). In addition, cue-elicited craving also shows no association with dependence level (Perkins 2009) or relapse (Perkins 2012), suggesting that drug cue reactivity is not associated with severity of addiction. The hierarchical account anticipates these null associations because all drug users should rapidly acquire comparable knowledge that drug cues signal the viability of drug-seeking behaviour. This means that drug cues should prime drug-seeking over baseline to a comparable extent irrespective of an individual’s level of drug use severity.

Higher AUDIT scores were associated with an overall increased preference for alcohol over food. Such associations between drug dependence severity and overall preferential drug choice have been consistently reported (Hogarth 2012; Hogarth and Chase 2011, 2012; Moeller et al. 2013, 2009) and suggest that drug dependence severity is underpinned by the ascription of greater relative value to drugs over other reinforcers (Ahmed 2010; Bickel et al. 2014; Heyman 2013; MacKillop 2016). By contrast, expectancy scores were not associated with an overall increase in alcohol choice.

The study reported a double dissociation: expectancy scores were associated with PIT but not overall alcohol choice, whereas AUDIT scores were associated with overall alcohol choice but not PIT. There was also no correlation between AUDIT and expectancy scores. The implication is that drug-seeking is governed by two independent processes (Cartoni et al. 2013; Hogarth 2012). Whereas the expected value of alcohol (indexed by AUDIT) determines the overall preference for alcohol, the expected viability of the alcohol-seeking response in the alcohol stimulus (indexed by expectancy scores) determines the alcohol PIT effect. This dual-process account of drug-seeking suggests that treatments must simultaneously address cue reactivity and expected drug value in order to improve therapeutic outcomes.

One unexpected result was that the magnitude of the food PIT effect was smaller in less dependent individuals. This was presumably due to food choice nearing maximal in blank trials in low-dependent individuals (approx. 80%), leaving little room for increase following food stimulus presentation. By contrast, alcohol choice peaked at around 60% in blank trials in more dependent individuals, and there was no reduction in the difference between alcohol and blank conditions as dependence increased, suggesting that the alcohol PIT effect was not similarly constrained by a ceiling effect.

In conclusion, the study used a biconditional PIT procedure to support a hierarchical learning account of drug cue reactivity. On this view, drug cues elicit an expectation that drug-seeking responses available in the current context are more effective, thus priming those responses. The study excluded the S-O-R account of cue reactivity which argues that drug expectancies directly elicit the drug-seeking responses with which they have been paired. Treatments which aim to reduce cue reactivity might therefore attempt to modify hierarchical knowledge that certain drug-seeking responses are viable in particular stimulus contexts. However, there remains the question as to what extent hierarchical knowledge, compared to simpler associative structures such as S-R or S-O-R, contributes to drug cue reactivity in the natural environment. Resolving this issue is crucial for determining which form of knowledge to target therapeutically.

## References

[CR1] Ahmed SH (2010). Validation crisis in animal models of drug addiction: beyond non-disordered drug use toward drug addiction. Neurosci Biobehav Rev.

[CR2] Babor TF, Higgins-Biddle JC, Saunders JB, Monteiro MG (2001) Audit. The Alcohol Use Disorders Identification Test (AUDIT): guidelines for use in primary care

[CR3] Bickel WK, Johnson MW, Koffarnus MN, MacKillop J, Murphy JG (2014). The behavioral economics of substance use disorders: reinforcement pathologies and their repair. Ann Rev Clin Psych.

[CR4] Bradfield LA, Balleine BW (2013). Hierarchical and binary associations compete for behavioral control during instrumental biconditional discrimination. J Exp Psychol: Anim Behav Processes.

[CR5] Carter BL, Tiffany ST (1999). Meta-analysis of cue-reactivity in addiction research. Addiction.

[CR6] Cartoni E, Puglisi-Allegra S, Baldassarre G (2013) The three principles of action: a Pavlovian-instrumental transfer hypothesis. Frontiers in Behavioral Neuroscience 7 10.3389/fnbeh.2013.00153 PMC3832805 24312025

[CR7] Cartoni E, Moretta T, Puglisi-Allegra S, Cabib S, Baldassarre G (2015). The relationship between specific Pavlovian instrumental transfer and instrumental reward probability. Front Psychol.

[CR8] Collins BN, Brandon TH (2002). Effects of extinction context and retrieval cues on alcohol cue reactivity among nonalcoholic drinkers. J Consult Clin Psychol.

[CR9] Colwill RM, Rescorla RA (1990). Evidence for the hierarchical structure of instrumental learning. Anim Learn Behav.

[CR10] Conklin CA, Tiffany ST (2002). Applying extinction research and theory to cue-exposure addiction treatments. Addiction.

[CR11] Declercq M, De Houwer J (2009). Evidence for a hierarchical structure underlying avoidance behavior. J Exp Psychol: Anim Behav Processes.

[CR12] Delamater AR (1996). Effects of several extinction treatments upon the integrity of Pavlovian stimulus-outcome associations. Learn Behav.

[CR13] Dickinson A, Bouton M, Fanselow M (1997). Bolles’s psychological syllogism. Learning, motivation and cognition.

[CR14] Dols M, Willems B, van den Hout M, Bittoun R (2000). Smokers can learn to influence their urge to smoke. Addict Behav.

[CR15] Field M, Duka T (2001). Smoking expectancy mediates the conditioned responses to arbitrary smoking cues. Behav Pharmacol.

[CR16] Gámez AM, Rosas JM (2005). Transfer of stimulus control across instrumental responses is attenuated by extinction in human instrumental conditioning. International Journal of Psychology & Psychological Therapy.

[CR17] Garbusow M, Schad DJ, Sommer C, Jünger E, Sebold M, Friedel E, Wendt J, Kathmann N, Schlagenhauf F, Zimmermann US, Heinz A, Huys QJM, Rapp MA (2014). Pavlovian-to-instrumental transfer in alcohol dependence: a pilot study. Neuropsychobiology.

[CR18] Hall G, Mitchell C, Graham S, Lavis Y (2003). Acquired equivalence and distinctiveness in human discrimination learning: evidence for associative mediation. J Exp Psychol Gen.

[CR19] Heyman GM (2013) Addiction and choice: theory and new data. Frontiers in Psychiatry 4 10.3389/fpsyt.2013.00031 PMC3644798 23653607

[CR20] Hogarth L (2012). Goal-directed and transfer-cue-elicited drug-seeking are dissociated by pharmacotherapy: evidence for independent additive controllers. J Exp Psychol: Anim Behav Processes.

[CR21] Hogarth L, Chase HW (2011). Parallel goal-directed and habitual control of human drug-seeking: implications for dependence vulnerability. J Exp Psychol: Anim Behav Processes.

[CR22] Hogarth L, Chase HW (2012). Evaluating psychological markers for human nicotine dependence: tobacco choice, extinction, and Pavlovian-to-instrumental transfer. Exp Clin Psychopharmacol.

[CR23] Hogarth L, Duka T (2006). Human nicotine conditioning requires explicit contingency knowledge: is addictive behaviour cognitively mediated?. Psychopharmacology.

[CR24] Hogarth L, Dickinson A, Wright A, Kouvaraki M, Duka T (2007). The role of drug expectancy in the control of human drug seeking. J Exp Psychol Anim Behav Process.

[CR25] Hogarth L, Retzler C, Munafò MR, Tran DMD, Troisi Ii JR, Rose AK, Jones A, Field M (2014). Extinction of cue-evoked drug-seeking relies on degrading hierarchical instrumental expectancies. Behav Res Ther.

[CR26] Hogarth L, Maynard OM, Munafò MR (2015). Plain cigarette packs do not exert Pavlovian to instrumental transfer of control over tobacco-seeking. Addiction.

[CR27] Jernigan D, Noel J, Landon J, Thornton N, Lobstein T (2017). Alcohol marketing and youth alcohol consumption: a systematic review of longitudinal studies published since 2008. Addiction.

[CR28] MacKillop J (2016) The behavioral economics and neuroeconomics of alcohol use disorders. Alcoholism: Clinical and Experimental Research Epub 2016 Mar 19 10.1111/acer.13004 PMC4846981 26993151

[CR29] Martinovic J, Jones A, Christiansen P, Rose AK, Hogarth L, Field M (2014). Electrophysiological responses to alcohol cues are not associated with Pavlovian-to-instrumental transfer in social drinkers. PLoS One.

[CR30] Mitchell CJ, De Houwer J, Lovibond PF (2009). The propositional nature of human associative learning. Behav Brain Sci.

[CR31] Moeller SJ, Maloney T, Parvaz MA, Dunning JP, Alia-Klein N, Woicik PA, Hajcak G, Telang F, Wang GJ, Volkow ND, Goldstein RZ (2009). Enhanced choice for viewing cocaine pictures in cocaine addiction. Biol Psychiatry.

[CR32] Moeller SJ, Beebe-Wang N, Woicik PA, Konova AB, Maloney T, Goldstein RZ (2013). Choice to view cocaine images predicts concurrent and prospective drug use in cocaine addiction. Drug Alcohol Depend.

[CR33] Perkins KA (2009). Does smoking cue-induced craving tell us anything important about nicotine dependence?. Addiction.

[CR34] Perkins KA (2012). Subjective reactivity to smoking cues as a predictor of quitting success. Nicotine Tob Res.

[CR35] Price KL, Saladin ME, Baker NL, Tolliver BK, DeSantis SM, McRae-Clark AL, Brady KT (2010). Extinction of drug cue reactivity in methamphetamine-dependent individuals. Behav Res Ther.

[CR36] Rescorla RA (1991). Associative relations in instrumental learning—the 18 Bartlett memorial lecture. Q J Exp Psychol B.

[CR37] Rescorla RA (1992). Associations between an instrumental discriminative stimulus and multiple outcomes. J Exp Psychol: Anim Behav Processes.

[CR38] Rescorla RA (1994). Control of instrumental performance by Pavlovian and instrumental stimuli. J Exp Psychol Anim Behav Process.

[CR39] Rosas JM, Paredes-Olay MC, García-Gutiérrez A, Espinosa JJ, Abad MJF (2010). Outcome-specific transfer between predictive and instrumental learning is unaffected by extinction but reversed by counterconditioning in human participants. Learn Motiv.

[CR40] Seabrooke T, Hogarth L, Mitchell C (2015) The propositional basis of cue-controlled reward seeking. The Quarterly Journal of Experimental Psychology: 1–40 10.1080/17470218.2015.1115885 26595818

[CR41] Stewart J, de Wit H, Eikelboom R (1984). Role of conditioned and unconditioned drug effects in self-administration of opiates and stimulants. Psychol Rev.

[CR42] Thewissen R, Snijders SJBD, Havermans RC, van den Hout M, Jansen A (2006). Renewal of cue-elicited urge to smoke: implications for cue exposure treatment. Behav Res Ther.

[CR43] Trask S, Bouton M (2014). Contextual control of operant behavior: evidence for hierarchical associations in instrumental learning. Learn Behav.

[CR44] Trick L, Hogarth L, Duka T (2011). Prediction and uncertainty in human Pavlovian to instrumental transfer. J Exp Psychol Learn Mem Cogn.

[CR45] Troisi JRI (2006). Pavlovian-instrumental transfer of the discriminative stimulus effects of nicotine and ethanol in rats. Psychol Rec.

[CR46] Wikler A (1984). Conditioning factors in opiate addiction and relapse. J Subst Abus Treat.

[CR47] de Wit S, Dickinson A (2009). Associative theories of goal-directed behaviour: a case for animal–human translational models. Psychological Research PRPF.

[CR48] Xue YX, Luo YX, Wu P, Shi HS, Xue LF, Chen C, Zhu WL, Ding ZB, Bao YP, Shi J, Epstein DH, Shaham Y, Lu L (2012). A memory retrieval-extinction procedure to prevent drug craving and relapse. Science.

